# Biochemical features of the Cry4B toxin of *Bacillus thuringiensis* subsp. *israelensis* and its interaction with BT-R_3_, a bitopic cadherin G-protein coupled receptor in *Anopheles gambiae*

**DOI:** 10.5281/zenodo.13169433

**Published:** 2024-08-02

**Authors:** Lee A. Bulla

**Affiliations:** 1 Department of Biological Sciences, The University of Texas at Dallas, Richardson, TX 75080-3021 USA.

## Abstract

**Introduction:**

The cadherin G-protein coupled receptor BT-R_3_ in the mosquito *Anopheles gambiae* is a single membrane-spanning α-helical (bitopic) protein that represents the most abundant and functionally diverse group of membrane proteins. Binding of the Cry4B toxin of *Bacillus thuringiensis* subsp. *israelensis* (Bti) to BT-R_3_ triggers a Mg2+-dependent signalling pathway in the mosquito that involves stimulation of G protein α-subunit, which subsequently launches a coordinated signalling cascade involving Na^+^/K^+^-ATPase. Described in this study is the behaviour of the Cry4B purified active protein toxin in solution relative to its protoxin predecessor produced by Bti as well as identification of the region within BT-R_3_ of *An. gambiae* to which the toxin binds.

**Materials and Methods:**

The relationship and behaviour of protoxin and toxin were ascertained *in vitro* by solubility studies in an alkaline environment like that of the mosquito larval midgut. To identify the specific toxin-binding site within BT-R_3_, the full-length coding sequence of the *bt-r3* gene was amplified and cloned in pENTR/D-TOTO and subcloned in pXINSECT-DEST38 resulting in recombinant pXINSECT-DEST38-*bt-r3*. Cytotoxicity was analysed using *Trichoplusia ni* High Five™ insect cells transfected with the pXINSECT-DEST38-*bt-r3* plasmid rendering them susceptible to the Cry4B toxin. Truncation mutational analyses, receptor-toxin binding studies and live cell experiments were used to elucidate the toxin-binding site in BT-R_3_.

**Results:**

The N-terminal half of the Cry4B protoxin was cleaved releasing active Cry4B toxin. The nontoxic C-terminal portion was degraded into small peptide fragments. The receptor BT-R_3_ contained a single toxin-binding site––a 106-amino acid polypeptide bounded by Ile1359 and Ser1464 (^1359^IS^1464^) localized in the 11th cadherin repeat of the receptor.

**Conclusions:**

The structural features of the toxin-binding site are critical to the specificity, selectivity and affinity of the active toxin and for the design and development of novel Bti-based biopesticides.

## Introduction

*Bacillus thuringiensis* subsp. *israelensis* (Bti) produces a complex of multiply shaped mosquitocidal parasporal crystals during sporulation that range in diameter from <0.1 to >1.5 μm [1]. Two proteins are present in the smallest parasporal crystals, the number of which increases proportionally with increasing crystal size. The largest crystals contain as many as seven proteins. The proteins are synthesised at different times during sporulation and are added to the developing crystals in a stepwise manner. They range in size from 28 to 72 kDa. Intact parasporal crystals are toxic to larvae of a variety of dipteran insects, including mosquitoes, black flies, horn flies and house flies [2-6]. Soluble crystals also kill adult mosquitoes, black flies, house flies and stable flies [7-10]. The plasmid pBtoxis (AL731825) of Bti encodes four parasporal Cry toxins (Cry4Aa, Cry4Ba, Cry10Aa and Cry11Aa) and three cytolytic (Cyt) proteins (Cyt1Aa, Cyt2Ba and Cyt1Ca) [11]. The designation Cry4B is used in the present study rather than Cry4Ba for consistency with previously published articles from my laboratory [12-15]. Cry toxins have variable specificity to different mosquitoes including *Aedes, Culex* and *Anopheles*, which transmit the most prominent mosquito-borne diseases [12]. The Cry4A and Cry11A toxins are highly effective against *Aedes* and *Culex* species but less so to *Anopheles*. Cry4B is most effective against *Anopheles* species but less so to *Aedes* and *Culex*. Cry10A has little or no effect on mosquitoes in general [13]. Cyt proteins are mammalian toxic components of parasporal crystals and are cytolytic to various invertebrate and mammalian cells [16-18]. One such toxin causes segmental reddened and edematous areas within the small intestine of rats with major lesions in the jejunum [17]. Notably, Cry proteins are not cytolytic. Sequence similarity of Cry toxins and Cyt toxins is statistically insignificant. A 28-kDa Cyt protein, representative of the Cyt toxin family, has been separated and purified chromatographically from the larger 72-kDa Cry toxin and shown to be minimally toxic to mosquito [18-21].

Of primary interest is the signalling receptor binding protein Cry4B toxin (UniProtKB P05519) encoded by the *cry4B* gene (1,923 bp) present on the Bti plasmid pBtoxis (127,923 bp) and its attendant signalling receptor BT-R_3_ (UniProtKB A0A1S4GG-P7) [14] located in the midgut epithelium of *An*. *gambiae*––an extremely efficient vector of the most dangerous human malaria protozoan parasite *Plasmodium falciparum*, *Wuchereria bancrofti*, a filarial nematode that causes lymphatic filariasis (or elephantiasis) manifested as severe lymphedema of the limbs and the O′nyong-nyong virus that causes rheumatic disease, primarily polyarthralgia. Earlier, the *cry4B* gene from Bti strain M1 (serotype H14) isolated from the Nile Delta Valley, Egypt, was cloned and expressed in several bacterial expression vectors [12,13]. Toxicity of recombinant Cry4B (LC_50_ = 2.0 x 10^2^ ng/ml) to *An. gambiae* is comparable to that of native Cry4B (1.8 x 10^2^ ng/ml) [12]. Recombinant Cry4A, Cry10A and Cry11A proteins are ineffective against *An. gambiae* [13].

Reported here are some of the key biochemical features of the Cry4B toxin and protoxin from which it is derived as well as the region within the BT-R_3_ molecule to which the toxin specifically binds. The Cry4B toxin is sequestered in a protoxin which occurs as a single repeating subunit in the parasporal crystals deposited alongside the endospore during sporulation. Following ingestion by *An. gambiae* larvae, the parasporal crystals are solubilised in an alkaline midgut environment and the protoxin is cleaved approximately in half by midgut proteases, generating active toxin. The remaining portion is degraded and nontoxic. The active Cry4B toxin is a first messenger that binds directly to BT-R_3_ (M_r_ ~ 195 kDa, 1735 aa), a bitopic (or single-pass) cadherin G protein-coupled receptor (GPCR), that activates the second messenger cAMP [12]. The univalent binding of Cry4B to BT-R_3_ triggers a Mg^2+^-dependent signalling pathway that activates adenylyl cyclase (AC) by its interaction with the α-subunit of the G_s_ protein (α_s_). Release of α_s_ from the heterotrimeric αβγ-G protein complex allows it to bind to and activate AC. The result is generation of cAMP which, in turn, activates the effector cAMP-dependent protein kinase A that stimulates Na^+^/K^+^-ATPase (NKA). NKA serves as a Na^+^/K^+^ pump to maintain proper gradients of Na^+^ and K^+^ ions across the cytoplasmic membrane. It also is a signal transducer that intensifies and accelerates the transmission of the initial message from the plasma membrane to the nucleus and influences various signal transduction cascades such as enhanced trafficking of BT-R_3_ to the cell surface. A greatly expanded receptor population on the cell surface attracts additional Cry4B toxin molecules. The consequent increased binding of toxins to receptors brings about cell death within 20-40 min of toxin exposure [12].

The structure of BT-R_3_ comprises four domains: Domain 1 (ectodomain, EC) composed of eleven ectodomain modules (EC1-EC11), each made up of β-barrel cadherin repeats connected one to another by interdomain linkers; Domain 2 (membrane-proximal extracellular domain, MPED); Domain 3 (single transmembrane α-helix domain, TM); Domain 4 (cytoplasmic domain, CYTO) [12-14]. Previously, it was determined that a region within EC modules 7-11 plus the MPED binds the Cry4B toxin [14]. To delineate the specific binding site within BT-R_3_, the full-length coding sequence of the *bt-r3* gene has been amplified and cloned in pENTR/D-TOTO and subsequently subcloned in pXINSECT-DEST38 resulting in recombinant pX-INSECT-DEST38-*bt-r3*. The pXINSECT-DEST38-*bt-r3* plasmid was used to transfect *Trichoplusia ni* High Five™ (H5) insect cells [22] which, consequently, became susceptible to the Cry4B toxin (M5 cells). Cytotoxicity was determined using stably transfected M5 cells. The present report shows that Cry4B binding to BT-R_3_ occurs in EC11 (^1355^YE^1466^) of Domain 1 adjacent to the MPED (Domain 2). Truncation mutant analysis of the EC along with binding and cytotoxicity inhibition assays demonstrate that the toxin-binding site (TBS) consists of a 106-amino acid polypeptide bounded by Ileu1359 and Ser1464 (^1359^IS^1464^). Determining active binding sites contributes to a better understanding of how Cry toxins like Cry4B interact with their attendant cadherin GPCRs and bring about death to mosquito vectors such as *An*. *gambiae* that transmit serious and deadly diseases.

Certainly, vector control is prominent in managing mosquito populations––Bti-based insecticides being among the most effective. Other promising approaches involve (i) vaccines such as RTS,S/AS01 and R21-Matrix M designed to stimulate the immune system, the mechanism of which is not well understood, (ii) various drugs directed at the main protozoan parasites, most of which are basically ineffective because of increased resistance of the parasites to the drugs, (iii) chemical insecticides, many of which are harmful to the environment, affect non-target organisms and are generally inefficient because of acquired insect resistance to the compounds and (iv) sterile insect techniques and transgenic mosquitoes whose validity has not been established in the wild. According to a World Health Organization (WHO) report published on August 23, 2023, “The global fight against malaria has stalled and requires massive investment as well as political leadership…”. Upon release of the report, Dr. Pedro Alonso, director of the WHO’s malaria programme, told a news conference in Geneva, Switzerland that “The world is at a crossroads. Historical progress that has been achieved over the last decade is clearly slowing down”. The WHO report warned “Despite huge progress in reducing malaria cases and deaths between 2000 and 2015, the last two years have witnessed the stalling of global progress.” The report further stated that a “massive concerted and coordinated action” is needed to eradicate the disease transmitted to humans through the bites of infected mosquitoes. I believe that Cry toxins such as Cry4B produced by Bti and cell surface targets such as BT-R_3_ are paramount to designing new and safer mosqui-tocides. Indeed, they have tremendous potential because Cry4B and constituent components can serve as selective targets or insecticide ligands and potentially act as (i) agonists which bind to a GPCR or to components of activated intracellular pathways, eliciting a biological response, (ii) antagonists which block the action of agonists, (iii) inverse agonists which cause action opposite to that of agonists and (iv) allosteric modulators which regulate GPCRs as the result of their binding to an effector molecule at a site other than the active site.

## Materials and Methods

### Purification of native and recombinant Cry4B toxin

Native Cry4B toxin was purified from parasporal crystals produced by the Bti strain M1 which was grown in T3 medium [23] containing tryptone, tryptose, yeast extract plus sodium phosphate, and magnesium and manganese salts at 30 °C until 90% of the cells were fully sporulated and the spores and parasporal crystals were released into the medium. Parasporal crystals were separated from spores by buoyant density centrifugation and washed in NaCl and dH_2_O for storage at –20 °C or for immediate use. Activation and purification of the Cry4B toxin was done as previously described [13]. Briefly, native crystals comprised of the 128-kDa protoxin were solubilised in alkaline (pH 10.5) buffer (50 mM Na_2_CO_3_ and 5 mM DTT) at room temperature for 2 h. The soluble protoxin was dialysed overnight at 4 °C in a buffer containing 50 mM Tris-HCl, 100 mM NaCl and 2 mM DTT, followed by trypsin treatment at 37 °C for 2h. The resulting activated 72-kDa toxin was then purified by anion exchange chromatography using an ÄKTA FPLC system and the appropriate fractions pooled, concentrated by centrifugation, quantified by the Bradford assay [24], and visualised by SDS-PAGE [25]. Aliquots of the purified Cry4B toxin were stored at –80 °C.

Recombinant Cry4B toxin was purified also as previously described [13]. Recombinant toxin was obtained using total RNA from Bti as a template for PCR. Primer design was based on the nucleotide sequence of the *cry4B* gene present in the pBtoxis plasmid [11]. Following PCR, the amplified *cry4B* gene was analysed in a 1% agarose gel and the amplicon was excised from the gel and purified. *Escherichia coli* TOPO10 and BL21 (DE3) strains (Invitrogen) were used for DNA manipulation and *cry4B* gene expression, respectively. pENTR/D-TOPO and pDEST-17 plasmids (Invitrogen) were used for cloning and expression, respectively, and were isolated and transformed into competent cells of *E. coli*. The purified *cry4B* gene was cloned in pEN-TR/D-TOPO and then subcloned in pDEST-17 and transformed into *E. coli* BL21 for expression. Using this approach, the *cry4B* gene was placed under the control of the T7 promoter. Recombinant protein purification was accomplished by growing the BL21 cells overnight on Luria Agar (LA) plates [26] containing ampicillin (100 μG/mL). Gene expression was induced by adding IPTG (0.5 nM) and harvesting the cells by centrifugation several hours later.

### Purification of native and recombinant BT-R3

Purification of native BT-R_3_ was done as previously described for BT-R_1_ [27, 28]. Briefly, brush border membrane vesicles (BBMV) were prepared from resected midguts of third-instar larvae of *An. gambiae*. Homogenised midgut tissue preparations were diluted with an equal volume of ice-cold 5 mM Tris-HCl (pH 7.4) containing 5 mM EDTA, 1 mM PMSF, 3 mM DTT and a cocktail of various protease inhibitors. Low-speed centrifugation (1,000 x g for 10 min) produced a pellet of heavier cellular debris and a second lighter fraction which was pelleted by ultracentrifugation (100,000 x g for 30 min). The resulting high-speed pellet contained essentially all BBMV. Enrichment of the BBMV proteins was accomplished by extracting the pellet in Triton X-114 which has a low cloud point at room temperature and produces separate detergent and aqueous phases, each of which was stored in liquid nitrogen at –80 °C.

Cloning and sequence analysis of BT-R_3_ was done according to previously described procedures that involve purification of total RNA from third-instar *An. gambiae* larvae [14], which was reverse-transcribed using appropriate primers. In short, the full-length coding sequence of *bt-r3* (5.2 kb) was amplified, cloned in the Gateway™ entry vector pENTR/D-TOPO (Invitrogen) and verified by Sanger sequencing. cDNA encoding the BT-R_3_ protein was cloned into pBluescript (Stratagene) and the BT-R_3_ coding sequence was subcloned in the Gateway™ destination vector pXINSECT-DEST38 (Invitrogen) by recombination with the pENTR/D-TOPO vector using LR clonase II (Invitrogen). Recombinant plasmids were transformed in an *E. coli* OmniMax2 T1 phage-resistant host grown on LA plates containing ampicillin (100 mg/mL) and chloramphenicol (34 mg/mL). Colonies that grew in ampicillin but not chloramphenicol were selected for further screening. Positive recombinant pX-INSECT-DEST38-BT-R_3_ constructs were confirmed by Sanger sequencing. An annotated amino acid sequence of BT-R_3_ deduced from the nucleotide sequence of BT-R_3_ cDNA cloned in pENTR-D-TOPO (GenBank KC310451) was reported previously [12].

### Expression of BT-R3 in High Five™ (H5) insect cells

A model system developed and proven in my laboratory [22] engages *T*. *ni* High Five^TM^ insect cells (BTI-TN-5B1-4) which can be readily transfected with the purified pXINSECT-DEST38-*bt-r3* plasmid using Cellfectin (Invitrogen). As specified previously [14], untransfected cells (H5) were grown as a monolayer in tissue culture flasks containing 5 mL of insect-Xpress medium (Lonza) containing gentamicin (10.0 μg/mL). H5 cells transfected with pXINSECT-DEST38-*bt-r3* were co-transfected with pBmA-neo, a neomycin-resistant plasmid, and were cultured in the insect-Xpress medium supplemented with G418 (Geneticin, 800 μg/mL). Geneticin-resistant H5 cells were sub-cultured for several generations to obtain transfected cells stably expressing full-length BT-R_3_ (designated M5 cells). Expression of *bt-r3* was achieved by RT-PCR using *bt-r3*-specific primers. Confirmation of *bt-r3* expression was done using plasma membranes prepared from H5 and M5 cells (1 x 106). Cells were lysed in CytoBuster protein extraction reagent (Novagen) and the lysates were centrifuged at high speed for 10 min at 4 °C and the pellets––predominantly plasma membrane––were dissolved in a membrane protein buffer (5M urea, 2M thiourea, 2% w/v CHAPS and 40 mM Tris-HCl). Cell extracts were always freshly prepared for detection of BT-R_3_. To determine Cry4B assimilation, cells were incubated with Cry4B at a specified concentration and time before preparation of cell extracts for measurement of toxin incorporation. Cell extracts were suspended in 100 μL of SDS-PAGE sample buffer, heated at 95 °C for 10 min and centrifuged for several minutes. Soluble membrane proteins were loaded on an SDS-polyacrylamide gel and resolved electrophoretically. Cry4B binding to BT-R_3_ was detected with anti-BT-R_3_ rabbit antiserum (1:2000) as primary antibody and horseradish peroxidase-conjugated goat-anti-rabbit antibody (1:10000) as secondary antibody. Membranes saturated with antibody were treated with ECL reagent for enhanced chemiluminescence and analysed with an Amer-sham ECL Plus Western Blotting Detection System.

### Construction of BT-R3 truncation mutants

BT-R_3_ cDNA was cloned into pBluescript (Strata-gene) as described for BT-R_1_ cDNA [29]. EC (Domain 1, amino acids 1-1466) and constituent fragments were cloned into pCITE (Novagen) by restriction enzyme digestion or PCR amplification using primers that encode each of the EC variants: EC-A (^1^MN^1320^), EC-B (^823^RE^1466^), EC-C (^1199^YE^1466^), EC-D (^1321^LE^1466^), EC-E (^1359^IS^1464^) and EC-F (^1467^ET^1582^). They all were cloned directionally into the expression plasmid vector pMal (Novagen), which includes the *malE* gene for production of fusion proteins in conjunction with maltose-binding protein (MBP) [30]. All plasmids were purified using plasmid preparation kits (Promega or Qiagen). The nucleotide sequence of the DNA inserts in the recombinant plasmids were verified by sequencing. Cloned PCR products were expressed in *E*. *coli* as MBP fusion proteins. MBP fusion greatly facilitated expression and purification of the peptides in the host bacteria and significantly enhanced their solubility. Expression and purification of the EC constructs were accomplished by selecting recombinant bacterial colonies grown overnight at 37 °C in 25 mL of Luria Broth (LB) [26] containing ampicillin (50 μg/mL) to obtain a starter culture. One liter of fresh LB was then inoculated with the starter culture and grown at 37 °C to an OD_600_ nm = 0.5. Recombinant protein production was induced by addition of IPTG to a final concentration of 100-500 μM. The bacteria were precipitated by centrifugation and resuspended in 20 mM Tris-HCl (pH 8.0) supplemented with 200 mM KCl, 5 mM BME, and 1 mM PMSF. Resuspended bacteria were lysed by sonication and the recombinant proteins were purified by ion-exchange chromatography followed by affinity chromatography with amylose beads as the refining step.

### Immunoligand blotting to detect toxin-bound BT-R3 fragments

Proteins were separated on SDS-polyacrylamide gradient gels (7.5–10%) and transferred to PVDF membranes. The membranes were blocked for 2 h at room temperature with Tris-HCl-buffered saline (pH 8) containing 5% non-fat dry milk powder, 5% glycerol and 0.1% Tween 20. For overlay assays, Cry4B toxin was added to the blocking buffer at a concentration of 5 nmol/L and incubated with the membranes for 2 h at room temperature. Cry4B binding to BT-R_3_ fragments was detected using anti-Cry4B antibody and visualised as described above for Cry4B binding to the BT-R_3_ molecule.

### Analysis of toxin-receptor interaction by gel filtration chromatography

Purified Cry4B toxin, MBP-EC11 protein and a mixture of these proteins at various molar ratios were applied to a Superdex 200 HR 10/30 column attached to an FPLC system (AP Biotech) equilibrated with Tris-buffered saline (pH 8.0) [30]. The concentrations of purified toxin and MBP-EC11 were determined by UV absorbance using calculated molar extinction coefficients. Eluates were monitored at 280 nm, and 0.5-mL fractions were collected for analysis. The proteins were examined by SDS-PAGE before and after gel filtration.

### Analysis of Cry4B toxin binding to EC fragments by amylose bead affinity chromatography

Binding of Cry4B toxin to the EC domains was determined using an affinity column-based assay [30]. MBP-linked EC fragments were bound to amylose matrix in small columns (approximately 150 μL) that were pre-equilibrated with a wash buffer composed of 20 mM Tris (pH 8.0) and 150 mM NaCl. The columns were washed twice with 1 mL of wash buffer to remove any unbound proteins and then were loaded with Cry4B toxin and washed twice with 1 mL of the wash buffer. Proteins were eluted in two aliquots of 400 μL by applying 10 mM maltose to the wash buffer. Samples from each elution step were analysed by SDS-PAGE.

### Cytotoxicity Studies

Toxicity of Cry4B to the transfected M5 cells was determined as previously described [14, 30] by growing the cells in 96-well plates (1 x 10^3^ cells/50 μL per well) to which fresh medium containing various concentrations (37.5-375 nM) of soluble Cry4B was added. Toxin-treated cells were incubated for 4 h at room temperature and cell death was quantified by trypan blue exclusion. Dead cells (blue-stained) and viable cells (unstained) were counted microscopically. Cytotoxicity was calculated by the ratio of dead cells/(dead cells + viable cells). Inhibition of Cry4B toxin action by soluble EC fragments was determined by testing their ability to inhibit toxin action by preincubating the fragments with Cry4B toxin (1:1 molar ratio) prior to addition to the growth medium. All experiments were done in triplicate and repeated six times.

## Results

### Domain Structure of the Cry4B Toxin

**Figure 1:**
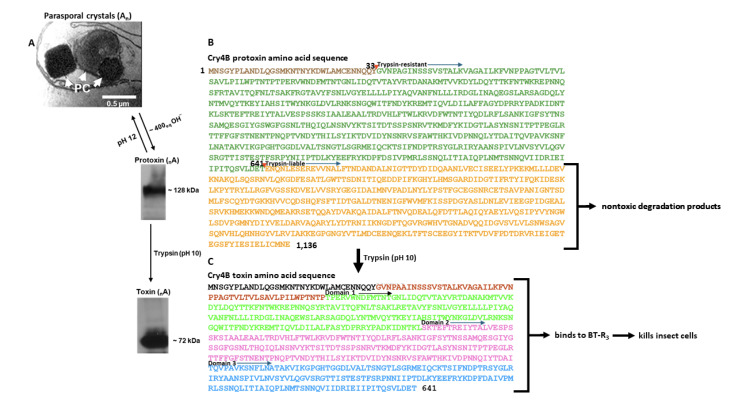
**Annotated amino acid sequences of the Cry4B protoxin and toxin deduced from the nucleotide sequence of the Cry4B protoxin gene of Bti (UniProtKB P05519).** A: Parasporal crystals produced by the Bti strain M1. B: Amino acid sequence of the protoxin (1ME1136) and the toxin (1MT641) embedded in the protoxin. The purple letters in Fig. 1B = N-terminal peptide 1MY33 which is released by trypsin (marked by a red arrowhead at 33YG34). The blue letters = trypsin-resistant polypeptide 34GT641 which is toxic. The N-terminal residue of the toxic, trypsin-resistant polypeptide is Gly34. The red letters = nontoxic segment (642EE1136). The N-terminus of the nontoxic segment is marked by a second red arrowhead (641TE642) which denotes trypsin liability (black arrow pointing downstream). C: Amino acid sequence of the toxin activated by trypsin in an alkaline buffer (pH 10) that binds to BT-R3, ultimately killing insect cells: Domain 1 (green letters, 84TL282); Domain 2 (pink letters, 283SI466); Domain 3 (blue letters, 467TT641). nontoxic portion (red letters, 642EE1136) is degraded to nontoxic products.

Figure 1 summarises the behaviour of purified Cry4B protoxin and toxin in solution. The cluster of parasporal crystals (A_n_) produced by Bti during sporulation is composed of many single repeating subunits that are dissociated (within minutes) in native conformation (_n_A) by mild alkali titration (Figure 1A). A subunit functionally acts as a protoxin (M_r_ ~ 128 kDa, 1136 aa) and is composed of an embedded toxin ^1^MT^641^ (Figure 1B, purple letters = N-terminal peptide ^1^MY^33^ released by trypsin; blue letters = trypsin-resistant polypeptide ^34^GT^641^) and a nontoxic segment ^642^EE^1136^ (Figure 1B, red letters,). The N-terminus of the toxic segment is marked by a red arrowhead (^33^YG^34^) which denotes trypsin resistance (see black arrow pointing downstream). The N-terminus of the nontoxic segment is marked by a red arrowhead (^641^TE^642^) which denotes trypsin liability (see black arrow pointing downstream). The fact that the single N-terminal residue methionine is present in quantitative yield indicates that the Cry4B protoxin is an intact product of translation. Trypsin activation in an alkaline buffer (pH 10) releases the toxin (Figure 1C, M_r_ ~ 72 kDa, 641 aa) from the nontoxic part which is degraded into smaller nontoxic fragments (Figure 1B). The activated toxin (_n_A), in turn, binds specifically to BT-R_3_ at a molar ratio of 1:1 resulting in cell death (Figure 1C).

The first reported crystal structure of Cry4B [31] is topologically like other Cry toxins including Cry1Aa and Cry3Bb [32-34] whose host range includes moths and beetles, respectively. The structural model consists of 558 residues (^84^TT^641^)––it lacks the first 83 residues (Figure 1C, black and red letters, ^1^MP^83^). A later X-ray crystallographic analysis [35] confirmed that of the first report but it too is not complete (^34^GT^641^)––it is missing the first 33 amino acid residues (Figure 1C, black letters, ^1^MY^33^). Nevertheless, cytotoxicity was not abolished by the absence of either of the N-terminal fragments. Unfortunately, the amino acid sequence reported (UniProtKB P05519) is that of protoxin, not toxin. For clarification, the Cry4B toxin is presented in relationship to its precursor, the Cry4B protoxin (Figures 1B and 1C). Protoxin *per se* is not toxic. When it is fed to mosquito larvae, its dissolution in the midgut releases the toxic portion which binds directly and specifically to BT-R_3_, killing the insect. The Cry4B toxin itself is composed of three domains (Figure 1C): Domain 1 (green letters, ^84^TL^282^), an α-helical bundle of five helices; Domain 2 (pink letters, ^283^SI^466^), a β-prism consisting of three antiparallel β-sheets; Domain 3 (blue letters, ^467^T-T^641^), a β-sandwich of two antiparallel β-sheets that form a jellyroll-like configuration. Trypsin treatment of the Cry4B toxin *in vitro* releases the N-terminal peptide ^1^MY^33^ (Figure 1C, black letters). Preliminary results (not shown) indicate that the N-terminal signal peptide most likely is contained within ^34^GP^83^ (Figure 1C, red letters). A peptidase recognition site has not been determined.

### Molecular structural organisation of BT-R3

**Figure 2:**
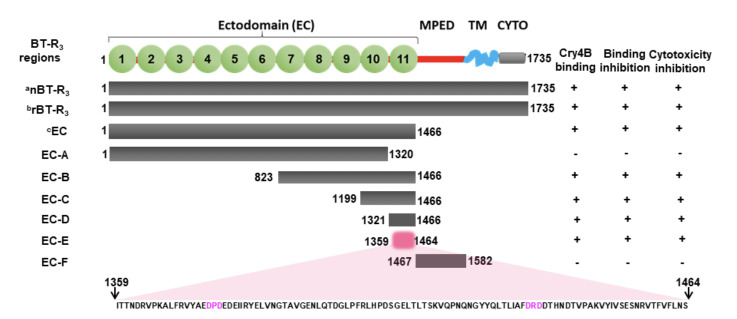
**Analysis of the Cry4B toxin-binding region in BT-R3 that mediates toxicity.** The schematic portrayal of the full-length BT-R3 receptor consists of 1735 amino acids which include the EC, MPED, TM and CYTO domains. Numbers flanking the regions indicate the position of amino acid residues. The EC (Domain 1) consists of 11 cadherin repeat modules indicated by the numbered green spheres. anBT-R3 refers to the native BT-R3 protein purified from BBMV of *A. gambiae*. brBT-R3 designates the full-length recombinant BT-R3 produced by High Five™ cells (M5) stably transfected with pXINSECT-DEST38-*bt-r3* as described in Materials and Methods. cEC mutant contains the complete ectodomain region; EC-A through EC-D are additional truncation mutants. The results of Cry4B toxin binding and inhibition of binding and cytotoxicity are summarised on the right and represent the mean ±SD of six experiments. Cry4B was administered at 79 nM, the concentration required to kill 50% of M5 cells [12]. EC, ectodomain; MPED, membrane-proximal extracellular domain; TM, single transmembrane α-helix domain; CYTO, cytoplasmic domain.

Figure 2 is a schematic depiction of the full-length BT-R_3_ receptor consisting of 1735 amino acids, which include the EC, MPED, TM and CYTO domains. The numbers flanking the regions indicate the position of the amino acid residues. The EC (Domain 1) comprises eleven cadherin repeat modules indicated by green spheres. Native BT-R_3_ protein (nBT-R_3_) was purified from BBMV of *A. gambiae* larvae. Full-length recombinant BT-R_3_ (rBT-R_3_) was deduced from the nucleotide sequence of BT-R_3_ cDNA cloned in pENTR-D-TOPO (GenBank KC310451) as previously reported [12]. The EC mutant contains the complete ectodomain region (^1^ME^1466^). EC-A through EC-F are additional truncation mutants. The additional EC variants collectively cover sequences between amino acids 1-1582 and are presented relative to the amino acid sequence of BT-R_3_ in Figure 2 and to the annotated amino acid sequence of BT-R_3_ [12].

### Identification and localisation of the Cry4B Toxin-binding Region in BT-R3

The results summarised to the right of the schematic in Figure 2 show that native and recombinant full-length BT-R_3_ proteins (^1^MF^1735^) and the EC domain (^1^ME^1466^) bind Cry4B toxin. Importantly, they also inhibited Cry4B toxin binding to BT-R_3_ as well as toxicity to M5 cells transfected with BT-R_3_ cDNA. Toxin binding to various fragments generated from the EC domain were further analysed by immunoligand blot analysis, affinity chromatography and gel filtration chromatography––the results of which are consolidated in Figure 2. As seen, Cry4B bound the EC and EC-B (^823^RE^1466^), EC-C (^1199^YE^1466^), EC-D (^1321^LE^1466^) and EC-E (^1359^IS^1464^) variants. Cry4B toxin binding to the native and recombinant BT-R_3_ proteins and to the EC domain was inhibited by those fragments containing EC11 (^1355^YE^1466^), as was cytotoxicity. Variant EC-A (^1^MN^1320^) which lies upstream of EC11, and EC-F (^1467^ET^1582^) which lies immediately downstream of EC11 did not bind toxin nor did they inhibit toxin binding and cytotoxicity. Variant EC-E is the smallest fragment in the EC11 module capable of binding the Cry4B toxin. This fragment which contains two Ca^2+^-binding motifs––DPD and DRD (red letters)–– is designated the TBS.

## Discussion

The bitopic cadherin G-protein coupled receptor BT-R_3_ in the mosquito *An*. *gambiae* represents a class of single membrane-spanning α-helical proteins within the cadherin family that regulate intercellular and intracellular signalling activities responsible for cell adhesion and signal transduction, respectively. Previous studies in my laboratory demonstrate that the Cry4B toxin is prominent among the various Cry toxins produced by Bti in exerting insecticidal action against *An. gambiae* [13] as well as several other mosquito species––vectors and non-vectors alike––and that such activity is mediated by BT-R_3_ [14] and related bitopic cadherin GPCRs. Moreover, a mechanism by which the Cry4B toxin kills host cells has been described [12]. It encompasses triggering a Mg^2+^-dependent signalling pathway that promotes stimulation of G protein α-subunit, which subsequently launches a coordinated signalling cascade involving NKA. In this study, the primary goal was to describe reliable procedures for rendering the protoxin and toxin in stable, soluble form and to define and map the Cry4B TBS within the *An. gambiae* receptor BT-R_3_. Recently, a protocol for analysing invertebrate bitopic cadherin G protein-coupled receptors that bind Cry toxins of *B. thuringiensis* was reported [15]. The report is a broad coverage of the experimental procedures used to analyse functional and structural features of cadherin GPCRs and the Cry toxins that they bind. The methods used in the present study are more narrowly focused on the relevant biochemical properties of the Cry 4B toxin and protoxin from which it is derived and on analysis of the region within the BT-R_3_ molecule to which the toxin specifically binds. The methods are repeatable and reproducible and include (i) purifcation of native and recombinant Cry4B toxin and BT-R_3_––protein purity being paramount for biophysical analyses such as X-ray crystallography, (ii) heterologous expression of BT-R_3_ in transfected High Five™ cells, (iii) gel filtration and amylose bead affinity chromatography of the Cry4B-BT-R_3_ and Cry4B-EC complexes and (iv) binding and cytotoxicity inhibition assays. The collective results summarised in Figure 2 provide conclusive evidence that the TBS is a 106-amino acid polypeptide bounded by Ile1359 and Ser1464 (^1359^IS^1464^) located in EC11, the proximal-most EC module in Domain 1 of BT-R_3_. Using a similar approach, it was previously shown that the TBS in the tobacco hornworm receptor, BT-R_1_, consists of a 94-amino acid polypeptide located in EC12 of Domain 1 of BT-R_1_ [36]. The binding affinity of the native and recombinant Cry4B toxins is extremely high (average K_d_ value of 36 nM), as is their capacity to kill larvae of *An. gambiae* (average LC_50_ = 1.9 x 10^2^ ng/ml) [12]. Likewise, the capacity to kill High Five™ cells transfected with BT-R_3_ cDNA (M5 cells) is remarkable. The LC_50_ value for Cry4B against M5 cells is 79.3 mM [12].

Among the salient features of cadherins is their role in determining cell patterning and preserving tissue architecture––cell-cell adhesion and signal transduction being critical to these functions. Cadherins (or calcium-dependent adherent proteins) require calcium for their activity, and the functional characteristics of cadherins are attributed to their Ca^2+^-dependent structural properties. The ectodomain of cadherins contains Ca^2+^- binding sites that stabilise their rodlike structure and preserve their adhesive function and molecular interactions. Case in point is the BT-R_3_ ectodomain (Domain 1) that contains ten predicted Ca^2+^-binding motifs that include DRD, DPD and DYD, one DRD motif in Domain 4 (CYTO) as well as two integrin recognition motifs, and RGD and LDV near EC1 and EC5, respectively [12]. The presence of the two Ca^2+^-binding motifs––DPD and DRD––in the TBS, which represents 20 percent of the Ca^2+^-binding motifs in the entire receptor molecule, is signifcant and most probably critical to its functionality. Ca^2+^-binding is essential to stabilising monomeric EC modules in appropriate conformation, i.e., *cis*dimer formation, thereby inhibiting fluctuations within the cadherin structure. Thermodynamic stabilisation of the monomer-monomer interactions is important in facilitating a variety of metabolic activities, including cell signaling [37]. Presumably, calcium binding to the TBS in BT-R_3_ helps maintain its correct structure and is involved in coordinating receptor-ligand interaction and the commensurate transmission of information into the interior of the cell. Earlier investigations of the lepidopteran receptor BT-R_1_ revealed that Ca^2+^ directly influences the structural and functional integrity of the receptor because removal of calcium ions by chelating agents promotes proteolytic cleavage of the BT-R_1_ ectodomain and loss of toxin binding [38]. Metal ions and free sulfhydryl groups are not involved in Cry1Ab binding to BT-R_1_ [39]. A similar scenario is true for Cry4B and BT-R_3_. Most likely, there is calcium-dependent protection of BT-R_3_ and other cadherin GPCRs as well. Characterisation of such a protection mechanism should provide further insight into the Ca^2+^-sensitive dynamics of the interaction of Cry toxins with their attendant GPCRs, which elicit a molecular signal rapidly transmitted into a cell. Moreover, Ca^2+^, acting as an endogenous ligand, appears to positively affect allosteric modulation of GPCR activity [40]. Ca^2+^ is an important allosteric modulator of Class B parathyroid hormone receptor signaling [41]. Whether Ca^2+^ (endogenous ligand) and Cry4B (exogenous ligand) work together in a coordinated manner to modulate GPCR function needs to be investigated. It may be that the Cry4B toxin directly targets the Ca^2+^ motifs in the TBS of BT-R_3_, activating the cell death pathway in *An. gambiae*. If so, it is quite feasible to use the Ca^2+^ pocket in the TBS of BT-R_3_ and related bitopic cadherin GPCR targets to realistically design alternative agents with new insecticidal profiles. Obviously, specificity is crucial to biorational insecticide design for any insect such as the mosquito *An. gambiae*, which is merely one species among ten quintillion individual insects that inhabit the world.

Additional research is necessary to develop more viable long-term Bti-based mosquitocidal products for field application. Direct application of Bti under field conditions to control mosquitoes has complications. To effectively control mosquito larvae, several factors must be considered. Their habitat may be lentic (standing water) or lotic (running water). Generally, most *Anopheles* larvae reside in both lentic and lotic environments whereas *Culex* and *Aedes* species are primarily lentic dwellers. Depending on the species, their feeding mode may be as filter-feeders (or plankton feeders), bottom feeders, scavengers, and predators, consuming particulate matter ranging in size from microscopic bacteria to clearly visible particles. Feeding mechanisms depend on organisation of their mouthparts, which remove suspended food particles from water and involve entrapment, ingestion directly into the mouth and bolus formation in the pharynx [42]. Clearly, field application of commercial formulations containing whole Bti bacteria is not simple and straightforward. The mode and timing of application, dosage (application) rate and the number of applications are major concerns, not to mention the cost involved. An additional matter is safety of the product. The general cytolytic and hemolytic activity of Cyt toxins inherent in native parasporal crystals of Bti is a potential threat to many nontarget species, including humans. Genetically engineering an organism to encode only the larvicidal activity associated with the 72-kDa Cry protein, which is not cytolytic, would alleviate any safety problems related to Cyt toxins. Introducing the *cry4B* gene into larval food sources, e.g., various eubacteria and cyanobacteria capable of replicating naturally [43], also would minimise repeated applications of Bti in the field. Furthermore, the animal and human population, as well as the environment, would be exposed to fewer chemical irritants and pollutants.

GPCRs represent the most important targets for insecticide discovery, and identifying ligand-binding sites in target GPCRs is central to a structure-based insecticide development program. GPCR activity can be measured straightforwardly by documenting the signalling pathways stimulated by these receptors. Signalling is initiated by the binding of an activated ligand (agonist) to the target receptor. This kind of binding activity can be assessed quantitively as can the kinetics of inhibitor binding to the receptor, i.e., antagonist binding [44]. A multitude of extracellular signal molecules (ligands) function at very low concentrations––dissociation constant K_d_ ≤ 10^-7^ M (<100 nM)––rendering them “high affinity”, and the receptors that bind them usually do so too with “high affinity” (affinity constant K_a_ ≥ 10^7^ liters/mole). As a point of reference, K_d_ ≡ 1 K_a_. Low affinity is revealed by a K_d_ value of greater than 10^-4^ (>100 μM); moderate affinity by a K_d_ value of 10^-4^-10^-7^ (100 μM-100 nM); and high affinity by a K_d_ value less than 10^-7^ (<100 nM). The Cry toxins produced by *B. thuringiensis* studied in my laboratory are orthosteric (target-specific) ligands that bind directly and tightly (K_d_ values in the low nanomolar range, i.e., <100 nM) [15] to a single binding site in insect GPCRs, preventing other molecules from interacting with them. An advantage of orthosteric ligands is that a low concentration of the ligand (Cry toxin) ensures exclusive binding to the signalling target receptor (BT-R), in the presence of other cell surface proteins, e.g., alkaline phosphatases, aminopeptidases, β-glucosidases and β-galactosidases, whose K_a_ values generally are <10^7^ liters/mole. Obviously, the Cry4B toxin has the shape, form and chemistry to match perfectly with the BT-R_3_ TBS, guaranteeing its selectivity and low, safe dosage/application rate for control of *An. gambiae*, thus reducing the impact of malaria and other important diseases. The molecular mode of action of all Cry toxins most likely is identical in that the activated toxin and the cognate receptor match like a key and lock. Differences at the molecular level between Cry toxins and their matching receptors provide a high degree of safety for this class of insecticides.

Malaria and other tropical diseases are extremely difficult, if not impossible, to eradicate. Indeed, the road to success is long and arduous. Therefore, a reasonable goal is reduction of vector populations to an acceptable level that possibly precludes human fatalities. Such an accomplishment can be achieved by integrated management in which Bti is involved. It is my hope that the information provided in this study contributes in a meaningful way to a better understanding of the mechanism of Cry protein toxicity and to the design and development of novel insecticides which will contribute to the future control of malaria.

Most certainly, “Freeing the world of malaria would be one of the greatest achievements in public health,” WHO Director-General Tedros Adhanom Ghebreyesus recently stated. “With new tools and approaches, we can make this vision a reality,” he concluded.

## Conclusions

The present study describes an overall experimental approach to examine the relevant biochemical properties and behaviour of soluble Bti Cry 4B protoxin and toxin *in vitro* and to define and analyse the region within the *An. gambiae* receptor BT-R_3_ that specifically binds the Cry4B toxin and kills insect cells transfected with the *bt-r3* gene. Further biochemical and biophysical characterisation of the Cry4B-BT-R_3_ complex in its natural conformational and functional state should provide a better understanding of invertebrate GPCR function in general and contribute to the structure-based development of new targets for mosquitocide discovery.

## References

[1] Lee SG, Eckblad W, Bulla LA Jr: Diversity of protein inclusion bodies and identification of mosquitocidal protein in *Bacillus thuringiensis* subsp. *israelensis*. Biochem. Biophys. Res. Commun. 1985, 126: 953-960. Doi: 10.1016/0006-291X(85)90278-5 2858207

[2] Tyrell DJ, Davidson LA, Bulla LA Jr, Ramoska WA: Toxicity of parasporal crystals of *Bacillus thuringiensis* subsp. *israelensis* to mosquitoes. Appl. Environ. Microbiol. 1979, 38:656-658. Doi: 10.1128/aem.38.4.656-658 44177 PMC243556

[3] Margalit J, Dean D: The story of *Bacillus thuringiensis* var. *israelensis* (B.t.i.). J. Am. Mosq. Control Assoc. 1985, 1: 1-7. 2906651

[4] Undeen AH, Nagel WL: The effect of *Bacillus thuringiensis* ONR-60A strain (Goldberg) on *Simulium* larvae in the laboratory. Mosq. News. 1978, 38: 524-527.

[5] Temeyer KB: Larvacidal activity of *Bacillus thuringiensis* subsp. *israelensis* in the dipteran *Haematobia irritans*. Appl. Environ. Microbiol. 1984, 47:952-955. Doi: 10.1128/aem.47.5.952-955 6742837 PMC240024

[6] Mwamburi LA, Laing MD, Miller R: Laboratory and field evaluation of formulated *Bacillus thuringiensis* var. *israelensis* as a feed additive and using topical applications for control of *Musca domestica* (Diptera: Muscidae) larvae in caged-poultry manure. Environmental Entomol. 2011, 40:52–58. Doi: 10.1603/EN09124 22182611

[7] Klowden MJ, Held GA, Bulla LA Jr: Toxicity of *Bacillus thuringiensis* subsp. *israelensis* to adult *Aedes aegypti* mosquitoes. Appl. Environ. Microbiol. 1983, 46: 312-315. Doi: 10.1128/aem.46.2.312-315 6625566 10.1128/aem.46.2.312-315.1983 PMC239378

[8] Klowden MJ, Bulla LA Jr: Oral toxicity of *Bacillus thuringiensis* subsp. *israelensis* to adult mosquitoes. Appl. Environ. Microbiol. 1984, 48:665-667. Doi: 10.1128/aem.48.3.665-667.1984 6149725 PMC241583

[9] Klowden MJ, Bulla LA Jr, Stoltz RL: Susceptibility of larval and adult *Simulium vittatum* (Diptera: Simulidae) to the solubilized parasporal crystal *Bacillus thuringiensis* subsp. *israelensis*. J. Med. Entomol. 1985, 22: 466-467. Doi: 10.1093/jmedent/22.4.466 4045942

[10] Wilton BE, Klowden MJ: Solubilized crystal of *Bacillus thuringiensis* subsp. *israelensis*: effect on adult house flies, stable flies (Diptera: Muscidae), and green lacewings (Neuroptera: chrysopidae). J. Am. Mosq. Control Assoc. 1985, 1:97-98. 3880220

[11] Berry CO, Neil S, Ben-Dov E, Jones AF : Complete sequence and organization of pBtoxis, the toxin-coding plasmid of *Bacillus thuringiensis* subsp. *israelensis*. Appl. Environ. Microbiol. 2002., 68: 5082–5095. Doi: 10.1128/AEM.68.10.5082-5095 12324359 10.1128/AEM.68.10.5082-5095.2002 PMC126441

[12] Liu L, Bulla LA Jr: Cell death signaling in *Anopheles gambiae* initiated by *Bacillus thuringiensis* Cry4B toxin involves Na+/K+ ATPase. Exp. Biol. Med. 2023 , 248 : 1191-1205 . Doi : 10.1177/15353702231188072 PMC10621475 37642306

[13] Ibrahim MA, Griko NB, Bulla LA Jr: The Cry4B toxin of *Bacillus thuringiensis* subsp. *israelensis* kills permethrin-resistant *Anopheles gambiae*, the principal vector of malaria. Exp. Biol. Med. 2013, 238:350-359. Doi: 10.1177/1535370213477973 23760000

[14] Ibrahim MA, Griko NB, Bulla LA Jr: Cytotoxicity of the *Bacillus thuringiensis* Cry4B toxin is mediated by the cadherin receptor BT-R3 of *Anopheles gambiae*. Exp. Biol. Med. 2013, 238:755-764. Doi: 10.1177/1535370213493719 23788176

[15] Liu L, Bulla LA Jr: Commentary: Analyzing invertebrate bitopic cadherin G protein-coupled receptors that bind Cry toxins of *Bacillus thuringiensis*. Comp. Biochem. Physiol. Part B. 2024, 110963. Doi: 10.1016/j.cbpb.2024.110963 38431088

[16] Pfannenstiel MA, Ross EJ, Kramer VC, Nickerson KW: Toxicity and composition of protease-inhibited *Bacillus thuringiensis* var. *israelensis* crystals. FEMS Microbiol. Lett. 1984, 21:29-32. Doi: 10.1111/j.1574-6968.1984.tb00182.x

[17] Thomas WE, Ellar DJ: *Bacillus thuringiensis* var. *israelensis* crystal δ-endotoxin: effects on insect and mammalian cells *in vitro* and *in vivo*. J. Cell Sci. 1983, 60:181-197. Doi: 10.1242/jcs.60.1.181 6874728

[18] Delécluse A, Charles J-F, Klier A, Rapoport G: Deletion by *in vivo* recombination shows that the 28-kilodalton cytolytic polypeptide from *Bacillus thuringiensis* subsp. *israelensis* is not essential for mosquitocidal activity. J. Bacteriol. 1991, 173: 3374-3381. Doi: 10.1128/jb.173.11.3374-3381.1991 1675212 PMC207948

[19] Mayes ME, Held GA, Lau C, Seely JC : Characterization of the mammalian toxicity of the crystal polypeptides of *Bacillus thuringiensis* subsp. *israelensis*. Fundam. Appl. Toxicol. 1989, 13: 310-322. Doi: 10.1016/0272-0590(89)90267-4 2792598

[20] Hurley JM, Lee SG, Andrews RE Jr, Klowden MJ, Bulla LA Jr: Separation of the cytolytic and mosquitocidal proteins of *Bacillus thuringiensis* subsp. *israelensis*. Biochem. Biophys. Res. Commun. 1985, 126: 961-965. Doi: 10.1016/0006-291X(85)90279-7 2858208

[21] Hurley JM, Bulla, LA Jr, Andrews RE Jr: Purification of the mosquitocidal and cytolytic proteins of *Bacillus thuringiensis* subsp. *israelensis*. Appl. Environ. Microbiol. 1987, 53:1316-1321. Doi: 10.1128/aem.53.6.1316-1321 3606108 10.1128/aem.53.6.1316-1321.1987 PMC203862

[22] Zhang X, Candas M, Griko NB, Rose-Young L, Bulla LA Jr: Cytotoxicity of *Bacillus thuringiensis* Cry1Ab toxin depends on specific binding of the toxin to the cadherin receptor BT-R1 expressed in insect cells. Cell. Death Differ. 2005, 12:1407-1416. Doi: 10.1038/sj.cdd.4401675 15920532

[23] Travers RS, Martin PAW, Reichelderfer CF: Selective process for efficient isolation of soil *Bacillus* spp. Appl. Environ. Microbiol. 1987, 53:1263-1266. Doi: 10.1128/aem.53.6.1263-1266.1987 16347359 PMC203852

[24] Bradford MM: A rapid and sensitive method for the quantitation of microgram quantities of protein utilizing the principle of protein-dye binding. Analyt. Biochem. 1976, 72:248-254. Doi: 10.1006/abio.1976.9999 942051

[25] Keeton TP, Bulla LA Jr: Ligand specificity and affinity of BT-R1, the *Bacillus thuringiensis* Cry1A toxin receptor from *Manduca sexta*, expressed in mammalian and insect cell cultures. Appl. Environ. Microbiol. 1997, 63:3419-3425. Doi: 10.1128/aem.63.9.3419-3425.1997 9292994 PMC168650

[26] Bertani G: Lysogeny at mid-twentieth century: P1, P2, and other experimental systems. J. Bacteriol. 2004, 186:595-600. Doi: 10.1128/JB.186.3.595-600 14729683 10.1128/JB.186.3.595-600.2004 PMC321500

[27] Vadlamudi RK, Ji TH, Bulla LA Jr: A specific binding protein from *Manduca sexta* for the insecticidal toxin of *Bacillus thuringiensis* subsp. *berliner*. J. Biol. Chem. 1993, 268:12334-12340. 8509372

[28] Keeton T, Francis BR, Maaty WSA, Bulla LA Jr: Effects of midgut-protein-preparative and ligand binding procedures on the toxin binding characteristics of BT-R1, a common high-affinity receptor in *Manduca sexta* for Cry1A *Bacillus thuringiensis* toxins. Appl. Environ. Microbiol. 1998, 64:2158-2165. Doi: 10.1128/aem.64.6.2158-2165.1998 9603829 PMC106293

[29] Vadlamudi RK, Weber E, Ji I, Ji TH, Bulla LA Jr: Cloning and expression of a receptor for an insecticidal toxin of *Bacillus thuringiensis*. *J. Biol. Chem.* 1995, 270: 5490–5494. Doi: 10.1074/jbc.270.10.5490 7890666

[30] Griko NB, Rose-Young L, Zhang X, Carpenter L : Univalent binding of the Cry1Ab toxin of *Bacillus thuringiensis* to a conserved structural motif in the cadherin receptor BT-R1. Biochemistry 2007, 46: 10001–10007. Doi: 10.1021/bi700769s 17696320

[31] Boonserm P, Davis P, Ellar DJ, Li J: Crystal structure of the mosquito-larvicidal toxin Cry4Ba and its biological implications. J. Mol. Biol. 2005, 348: 363-382. Doi: 10.1016/j.jmb.2005.02.013 15811374

[32] Grochulski P, Masson L, Borisova S, Pusztai-Carey M : *Bacillus thuringiensis* CryIA(a) insecticidal toxin crystal structure and channel formation. J. Mol. Biol. 1995, 254:447-464. Doi: 10.1006/jmbi.1995.0630 7490762

[33] Galitsky N, Cody V, Wojtczak A, Ghosh D : Structure of the insecticidal bacterial δ-endotoxin Cry3Bb of *Bacillus thuringiensis*. Acta. Crystallogr. Sect. D. Biological Crystallography. 2001, 57: 1101-1109. Doi: 10.1107/s0907444901008186 11468393

[34] Li JD, Carroll J, Ellar DJ: Crystal structure of insecticidal delta-endotoxin from *Bacillus thuringiensis* at 2.5 A resolution. Nature. 1991, 353:815-821. Doi: 10.1038/353815a0 1658659

[35] Thamwiriyasati N, Angsuthanasombat C, Chen C-J: Crystal structure of CRY4BA-R203Q TOXIN. 2020, Protein Data Bank ID: 4MOA. Doi: 10.2210/pdb4moa/pdb

[36] Liu L, Wilcox XE, Fisher AJ, Boyd SD : Functional and structural analysis of the toxin-binding site of the cadherin G‑protein-coupled receptor, BT‑R1, for Cry1A toxins of *Bacillus thuringiensis*. Biochemistry. 2022, 61:752-766. Doi: 10.1021/acs.biochem.2c00089 35438971

[37] Cailliez F, Lavery R: Cadherin mechanics and complexation: the importance of calcium binding. Biophysical Journal. 2005, 89:3895-3903. Doi: 10.1529/biophysj.105.067322 16183887 PMC1366956

[38] Candas M, Francis BR, Griko NB, Midboe EG, Bulla LA Jr: Proteolytic cleavage of the developmentally important cadherin BT-R1 in the midgut epithelium of *Manduca sexta*. Biochemistry 2002, 41: 13717-13724. Doi: 10.1021/bi026323k 12427034

[39] Francis BR, Bulla LA Jr: Further characterization of BT-R1, the cadherin-like receptor for Cry1Ab toxin in tobacco hornworm (*Manduca sexta*) midguts. Insect Biochem. Molec. Biol. 1997, 27: 541-550. Doi: 10.1016/s0965-1748(97)00029-5 9304795

[40] Zarzycka B, Zaidi SA, Roth BL, Katritch V: Harnessing ion-binding sites for GPCR pharmacology. Pharmacol. Rev. 2019, 71: 571–595. Doi: 10.1124/pr.119.017863 31551350 PMC6782022

[41] White AD, Fang F, Jean-Alphonse FG, Clark LJ : Ca2+ allostery in PTH-receptor signaling. Proc. Natl. Acad. Sci. USA. 2019, 116: 3294–3299. Doi: 10.1073/pnas.1814670116 30718391 PMC6386702

[42] Merritt RW, Dadd, RH, Walker ED: Feeding behavior, natural food, and nutritional relationships of larval mosquitoes. Annu. Rev. Entomol. 1992, 37: 349-374. Doi: 10.1146/annurev.en.37.010192.002025 1347208

[43] Raymond KC, Wabiko H, Faust RM, Bulla LA Jr: Transfer of the *Bacillus thuringiensis israelensis* mosquitocidal toxin gene into mosquito larval food sources. In: de Barjac H, and Sutherland DJ, editors. Bacterial Control of Mosquitoes & Black Flies: Biochemistry, Genetics & Applications of Bacillus thuringiensis israelensis and Bacillus sphaericus. Springer Science & Business Media. 2012, 349 pp.

[44] Hoare SRJ: Analyzing kinetic binding data. In: Markossian S, Grossman A, Arkin M , editors. Assay Guidance Manual [Internet]. Bethesda (MD): Eli Lilly & Company and the National Center for Advancing Translational Sciences; 2004-.2021. 40 pp. https://tinyurl.com/2yfufj8n 33852262

